# Transcription factor stoichiometry, motif affinity and syntax regulate single-cell chromatin dynamics during fibroblast reprogramming to pluripotency

**DOI:** 10.1101/2023.10.04.560808

**Published:** 2023-10-21

**Authors:** Surag Nair, Mohamed Ameen, Laksshman Sundaram, Anusri Pampari, Jacob Schreiber, Akshay Balsubramani, Yu Xin Wang, David Burns, Helen M Blau, Ioannis Karakikes, Kevin C Wang, Anshul Kundaje

**Affiliations:** 1 Department of Computer Science, Stanford University, Stanford, CA, USA; 2 Department of Cancer Biology, Stanford University, Stanford, CA, USA; 3 Cardiovascular Institute, Stanford University, Stanford, CA, USA; 4 Department of Dermatology, Stanford University, Stanford, CA, USA; 5 Program in Epithelial Biology, Stanford University, Stanford, CA, USA; 6 Department of Genetics, Stanford University, Stanford, CA, USA; 7 Baxter Laboratory for Stem Cell Biology, Stanford University, Stanford, CA, USA; 8 Department of Microbiology and Immunology, Stanford University, Stanford, CA, USA; 9 Department of Cardiothoracic Surgery, Stanford University, Stanford, CA, USA; 10 Veterans Affairs Palo Alto Healthcare System, Palo Alto, CA, USA

## Abstract

Ectopic expression of *OCT4*, *SOX2*, *KLF4* and *MYC* (OSKM) transforms differentiated cells into induced pluripotent stem cells. To refine our mechanistic understanding of reprogramming, especially during the early stages, we profiled chromatin accessibility and gene expression at single-cell resolution across a densely sampled time course of human fibroblast reprogramming. Using neural networks that map DNA sequence to ATAC-seq profiles at base-resolution, we annotated cell-state-specific predictive transcription factor (TF) motif syntax in regulatory elements, inferred affinity- and concentration-dependent dynamics of Tn5-bias corrected TF footprints, linked peaks to putative target genes, and elucidated rewiring of TF-to-gene cis-regulatory networks. Our models reveal that early in reprogramming, OSK, at supraphysiological concentrations, rapidly open transient regulatory elements by occupying non-canonical low-affinity binding sites. As OSK concentration falls, the accessibility of these transient elements decays as a function of motif affinity. We find that these OSK-dependent transient elements sequester the somatic TF AP-1. This redistribution is strongly associated with the silencing of fibroblast-specific genes within individual nuclei. Together, our integrated single-cell resource and models reveal insights into the cis-regulatory code of reprogramming at unprecedented resolution, connect TF stoichiometry and motif syntax to diversification of cell fate trajectories, and provide new perspectives on the dynamics and role of transient regulatory elements in somatic silencing.

## Introduction

Ectopic expression of the Yamanaka transcription factors (TFs) *POU5F1* (*OCT4*), *SOX2*, *KLF4* and *MYC* (OSKM) in differentiated cells can reprogram them into induced pluripotent stem cells (iPSCs) (Takahashi and Yamanaka, 2006). Similar to embryonic stem cells (ESCs), iPSCs self-renew and differentiate into any cell type of the body. iPSCs offer unprecedented opportunities for regenerative medicine, as they can be derived from adult patients to recapitulate diverse patient-specific cell types and tissues for transplantation, organoid generation, drug screening, and disease modeling (Singh *et al.*, 2015). However, iPSC technology still faces numerous challenges, including low reprogramming efficiency, variable quality, genomic instability, tumorigenicity, and incomplete silencing of the starting cell state (Schlaeger *et al.*, 2015; Buckberry *et al.*, 2023). A fundamental understanding of the regulatory control of cellular identity and cell state transitions over the course of reprogramming is critical to address these challenges.

Pioneering studies characterizing molecular features of fibroblast reprogramming have shown that overexpression of OSKM in somatic cells leads to extensive opening of closed chromatin, initiating multiple trajectories of cell state transitions, some of which culminate in successfully reprogrammed iPSCs (Soufi *et al.*, 2012; Polo *et al.*, 2012; Deng *et al.*, 2021). These studies employed bulk transcriptome and chromatin profiling experiments in heterogeneous cell populations or explicitly sorted reprogramming intermediates based on expression of surface markers of fibroblasts and iPSCs (Polo *et al.*, 2012; Li *et al.*, 2017; Chronis *et al.*, 2017; Knaupp *et al.*, 2017; Cacchiarelli *et al.*, 2015). These study designs have the potential to mask heterogeneity and preclude examination of a progression of intermediate states during reprogramming progression.

More recently, single-cell studies have enabled characterization of reprogramming intermediates at higher resolution (Guo *et al.*, 2019; Xing *et al.*, 2020; Liu *et al.*, 2020; Schiebinger *et al.*, 2019). Single-cell expression and chromatin profiling across human and mouse fibroblast reprogramming time courses show that somatic genes and regulatory elements are silenced early on, while pluripotency-associated genes and regulatory elements are progressively activated in phases over a span of 3 to 4 weeks (Cacchiarelli *et al.*, 2015; Chronis *et al.*, 2017; Li *et al.*, 2017; Knaupp *et al.*, 2017; Xing *et al.*, 2020; Liu *et al.*, 2020). These critical transitions towards a pluripotent cell fate are accompanied by widespread transient activity characterized by temporally restricted activation of genes and regulatory elements that are otherwise dormant in fibroblasts and iPSCs. While these studies have significantly advanced our understanding of regulatory dynamics of gene expression and control of reprogramming, the role of transient genes and regulatory elements in reprogramming remains elusive (Deng *et al.*, 2021).

Complementary approaches have demonstrated that the stoichiometry of ectopic OSKM at initiation and throughout the overexpression time course are critical determinants of the initiation, progression and efficiency of reprogramming (Papapetrou *et al.*, 2009; Carey *et al.*, 2011; Yamaguchi *et al.*, 2011; Buganim *et al.*, 2013; Takahashi and Yamanaka, 2016; Ilia *et al.*, 2023). However, it remains unclear how the interplay between OSKM stoichiometry and cis-regulatory sequences determines their quantitative occupancy at target sites, and how their combinatorial binding directs chromatin accessibility, gene expression dynamics, and ultimately reprogramming outcomes.

Recently, neural networks have emerged as state-of-the-art predictive models of regulatory DNA (Avsec, Agarwal, *et al.*, 2021; Zhou *et al.*, 2019; Chen *et al.*, 2022; Eraslan *et al.*, 2019; Avsec, Weilert, *et al.*, 2021; Zhou, 2022). These models are trained to accurately map regulatory DNA sequences to associated experimental profiles of TF binding, chromatin accessibility and histone marks in cellular contexts of interest. The models learn hierarchical layers of *de novo* sequence pattern detectors that can encode sequence motifs and their higher-order syntax. Interpretation of these models has revealed novel insights into the cis-regulatory code of TF binding including sequence preferences and affinity landscapes of individual TFs (Avsec, Weilert, *et al.*, 2021; Alexandari *et al.*, 2023), soft motif syntax mediated TF cooperativity (Avsec, Weilert, *et al.*, 2021; de Almeida *et al.*, 2022), and effects of sequence variation and repeats (Horton *et al.*, 2023; Alexandari *et al.*, 2023; Avsec, Agarwal, *et al.*, 2021; Chen *et al.*, 2022). While neural networks have been used to dissect the sequence basis of chromatin accessibility of diverse cell types (Maslova *et al.*, 2020; Kim *et al.*, 2021; Janssens *et al.*, 2022; Ameen *et al.*, 2022), they have yet to be used to decipher cis-regulatory drivers of quantitative chromatin dynamics from single-cell profiling across continuous cell state transitions during reprogramming.

In this study, we profiled chromatin accessibility and gene expression at single-cell resolution across a densely sampled time course of OSKM induced reprogramming of human fibroblasts. To investigate the earliest phase of reprogramming, we also jointly profiled single-nucleus chromatin accessibility and expression on the first and second days post-induction. We integrated both data modalities to provide a high-resolution view of the dynamic chromatin and transcriptomic landscape of intermediate cell states associated with distinct stoichiometry of OSKM that span a primary reprogramming trajectory and multiple, distinct off-target trajectories. To unravel the cis-regulatory sequence lexicon of chromatin dynamics, we trained and interpreted convolutional neural networks (called ChromBPNet) that accurately predict cell-state-specific ATAC-seq profiles at base-resolution from the underlying DNA sequence. We systematically annotated cell-state-specific predictive TF motif syntax in all regulatory elements, inferred dynamics of Tn5-bias corrected TF footprints as a function of TF concentration and motif affinity, linked dynamic elements to putative target genes, and elucidated rewiring of TF-to-gene cis-regulatory networks across cell state transitions in reprogramming. Our models reveal that early in reprogramming, OSK, at supraphysiological concentrations, rapidly open transient regulatory elements by occupying constellations of low-affinity binding sites, including non-canonical forms of the OCT-SOX heterodimer. The decay rate of accessibility of these transient elements is a function of motif affinity and OSK concentration. Additionally, these transient elements also contain occupied binding sites of somatic TFs such as AP-1. These binding sites are distinct from those found in fibroblast-specific regulatory elements and appear to sequester somatic TFs away from their fibroblast-specific sites. We identify a strong quantitative association between the sequestration of somatic TFs and silencing of fibroblast-specific genes, supporting a “repression-by-theft” hypothesis (Chronis *et al.*, 2017; Knaupp *et al.*, 2017; Deng *et al.*, 2021). Our integrated single-cell resource and associated machine learning models enable us to dissect the cis-regulatory code of reprogramming at unprecedented resolution, and connect TF stoichiometry, motif affinity and syntax to a diversity of cell fate trajectories. Together, our study provides a fresh perspective and new insights into the dynamics and role of transient regulatory elements in the silencing of somatic gene expression programs and concomitant activation of pluripotency programs.

## Single-cell transcriptomes and chromatin profiles of fibroblast reprogramming reveal diverse cell fate trajectories with continuous cell state transitions

**Fig 1:**
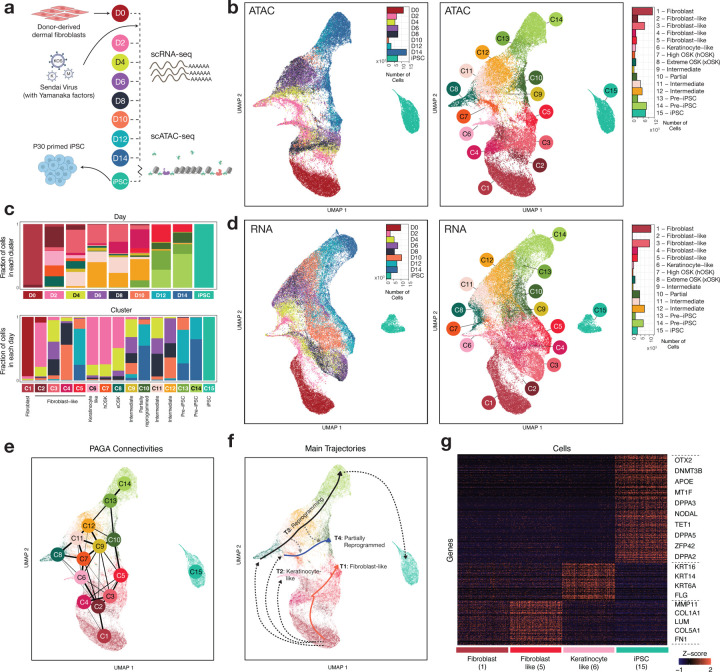
Single-cell chromatin and transcriptomic profiling of fibroblast reprogramming **a)** Schematic of experimental design **b)** UMAP of single-cell ATAC-seq cells labeled by timepoint (left) and cell state identity (right) **c)** Barplots showing cluster-wise composition of scATAC-seq cells in each day (top), and day-wise composition of each cluster (bottom). The day-wise plots are normalized to account for differences in the total number of cells from each day. **d)** UMAP of single-cell RNA-seq cells labeled by timepoint (left) and cell state identity (right) **e)** PAGA connectivity graph derived from scATAC-seq data **f)** Key trajectories of cells overlaid on scATAC-seq UMAP **g)** Marker genes for the start state (Fibroblast) and multiple putative end states

We overexpressed the reprogramming factors OSKM using a non-integrative Sendai virus system in primary dermal fibroblasts. To induce iPSC reprogramming, fibroblasts were transduced by Sendai viruses at MOI=5-5-3 (KOS MOI=5, *hMYC* MOI=5, and *hKLF4* MOI=3). We then harvested cells at D2, D4, D6, D8, D10, D12, and D14, as well as the starting population of fibroblasts (D0) and endpoint of iPSCs at passage 30, and processed them for scRNA-seq and scATAC-seq using the Chromium 10X platform (Fig 1a, Methods).

The scATAC-seq data yielded 62,599 cells that passed quality control metrics (Fig X1a, Methods). We used SnapATAC’s implementation of diffusion maps to perform dimensionality reduction on scATAC-seq data from all time points, followed by a 2-dimensional uniform manifold approximation and projection (UMAP) (Fang *et al.*, 2021; McInnes *et al.*, 2018). We used the Leiden algorithm to cluster cells based on their scATAC-seq profiles into 15 cell states (C1-C15) (Fig 1b). The initial day 0 population of fibroblasts and final population of iPSCs formed well separated clusters. On the other hand, samples from days 2–14 were composed of varying proportions of cells from different transient sub-populations, suggesting a continuum of cell states across the reprogramming process (Fig 1b,c). For cells from days 2–14, clustering provides approximate, representative cell state landmarks that simplify exposition and increase power for downstream analyses. We identified 525,835 scATAC-seq peaks representing putative cis-regulatory elements over all 15 cell states.

The scRNA-seq data yielded 59,378 high-quality cells (Fig X1b, Methods). We used Seurat for preprocessing the scRNA-seq data, followed by principal components analysis and UMAP (Stuart *et al.*, 2019) (Fig 1d). Cells from days 2–14 exhibited substantial heterogeneity of gene expression profiles, in agreement with the scATAC-seq analysis.

Next, we estimated the similarity of each cell’s chromatin and expression landscape to that of canonical fibroblast cells and embryonic stem cells respectively. As expected, we observed an early loss of somatic identity and a gradual gain of pluripotency (Fig S1a), in line with previous observations in mouse reprogramming systems (Li *et al.*, 2017). The chromatin-derived temporal dynamics of somatic to pluripotent identity transitions were broadly consistent with those obtained from analogous transcriptome derived similarity scores (Fig S1b). Reduction in expression of fibroblast markers like *COL1A1* and *FN1* was accompanied by reduced chromatin accessibility in and around their gene bodies, while changes in chromatin landscape often preceded expression of reprogramming-linked genes such as *NANOG* and *CDH1* (Fig S1c).

We aligned cells from the scRNA-seq and scATAC-seq experiments in a joint CCA embedding in order to transfer scATAC-seq derived cell state labels to cells in the RNA compendium (Fig S1d,e, Methods). In many cases, unambiguous assignment of scATAC-seq cell state labels to cells in the RNA compendium was not possible. Cells from the RNA compendium were often assigned high probabilities of matching two or more neighboring scATAC-seq derived cell states, again suggesting a smooth continuum of cell state transitions (Fig S1f,g). Next, to identify putative regulatory peak-gene associations, we calculated the correlation of scATAC-seq coverage and scRNA-seq expression between all pairs of scATAC-seq peaks and nearby genes across all aligned cells. 156,016 statistically significant (FDR < 0.001, t-test) peak-gene associations passed an absolute correlation threshold of 0.45, with a median 7 peaks per gene (max 97, min 0) for the top 2000 most variable genes, and 1 gene per peak (max 7, Table S1).

We used the PAGA abstraction graph on the scATAC-seq data to reveal relationships between cell states in terms of enriched connectivity based on cell similarity (Wolf *et al.*, 2019) (Fig 1e, Methods). Cell states (C6–8) primarily comprising day 2 cells were disconnected from the initial fibroblast population (C1), suggesting that ectopic overexpression of reprogramming factors dramatically shifts the chromatin and transcriptomic landscapes within two days and lends an appearance of cells “teleporting” early on in the time course (Polo *et al.*, 2012). From day 2 onwards, the PAGA graph showed relatively strong interconnectedness between a continuum of intermediate cell states (C6-C12) leading up to Pre-iPSCs (C13,C14). Finally, the iPSC cells (C15) formed a disconnected cluster, suggesting substantial differences between Pre-iPSCs (C13, 14) and iPSCs acquired over 30 rounds of passaging.

Diffusion based pseudotime inference identified four main trajectories of cell state transitions: two off-target trajectories terminating in stalled fibroblast-like cells (T1) and keratinocyte-like cells (T2), a primary reprogramming trajectory that terminates in iPSC cells (T3), and a parallel trajectory that ends in partially reprogrammed cells at day 14 (T4) (Fig 1f, Fig S1h,i, Methods) (Haghverdi *et al.*, 2016). Starting from a homogeneous population of dermal fibroblast cells at day 0 (C1), heterogeneity arises immediately after OSKM induction as many cells appear to fail to initiate reprogramming (T1, T2). Some cells remain in fibroblast-like states throughout the time-course. This fibroblast-like trajectory (T1) activates an interferon antiviral response characterized by expression of oligoadenylate synthetases (*OAS*) genes (Fig X2a,b,c (Gene Set 4), X2d) (Melchjorsen *et al.*, 2009; Sadler and Williams, 2008), and culminates in cells (C2-C5) that express higher levels of fibroblast markers such as *COL1A1* and *FN1* than the starting fibroblast population (Fig 1g). Other fibroblast cells transition (T2) into a terminal population of keratinocyte-like cells (C6) that express keratinocyte markers such as *KRT14*, *KRT16* and *FLG* (Gazel *et al.*, 2003).

**Fig 2:**
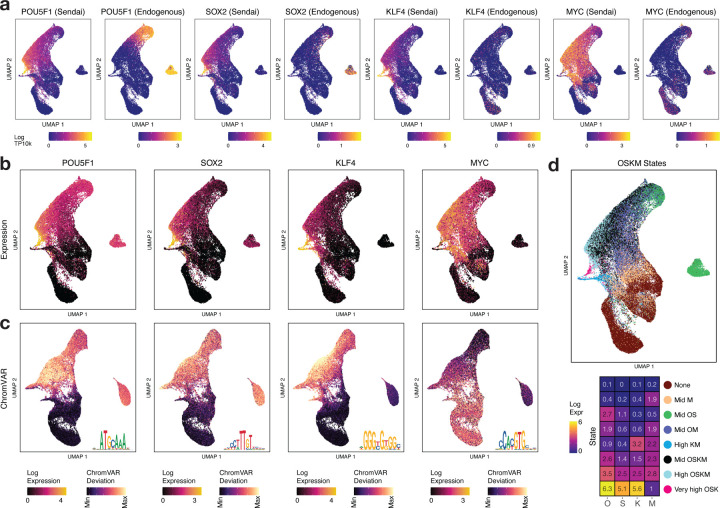
Stoichiometry and temporal dynamics of OSKM expression **a)** Estimated exogenous (Sendai-derived) and endogenous expression of OSKM **b)** Total expression of OSKM factors overlaid on scRNA-seq UMAP **c)** ChromVAR deviation scores of OSKM motifs overlaid on scATAC-seq UMAP. Motif shown as inset. **d)** States derived from k-means clustering of OSKM expression: scRNA-seq cells labeled by OSKM state (top) and centroid expression values for each state (bottom)

We devised a method to deconvolve endogenous and exogenous Sendai transcripts of OSKM (Fig 2a, Methods). Day 2 cells in clusters C7 and C8 that appear to initiate reprogramming, show high and extreme levels of Sendai OSK expression relative to endogenous OSK expression in iPSCs (Fig 2a) and were hence labeled as High OSK (hOSK) and Extreme OSK (xOSK) respectively. Cells in the reprogramming trajectory (T3) starting from the xOSK state (C8) proceed through intermediate states (C11, 12) and Pre-iPSC states (C13, 14) before terminating in the iPSC state (C15) after 30 rounds of passaging. iPSC cells express canonical pluripotency-associated genes such as *OTX2*, *TET1* and *ZFP42* (Fig 1g). In contrast, cells along the partially reprogrammed trajectory (T4) starting from the hOSK state (C7) proceed through intermediate state C9 and finally end in the partially reprogrammed state (C10). Partially reprogrammed cells failed to activate endogenous *OCT4* expression, in stark contrast to Pre-iPSC cells (Fig 2a). Furthermore, transgene expression levels were higher in states along the reprogramming trajectory (T3) compared to the partially reprogrammed trajectory (T4). Under the assumption that transgene levels decrease over time due to dilution of Sendai virus vectors (Fusaki *et al.*, 2009), the gradient of transgene expression suggests that some cells fall off the primary reprogramming trajectory (T3) into the partially reprogrammed trajectory (T4) (Fig 1f gray dotted arrows, Fig S1j, Methods). This is consistent with the observation that premature withdrawal of OSKM expression can stall the progress of cells in a partially reprogrammed state (Tanabe *et al.*, 2013).

In summary, the single-cell landscape of gene expressions and chromatin accessibility post OSKM induction highlight a continuum of cell state transitions involving a primary trajectory to successful reprogramming, and several off-target trajectories involving cells that fail to either initiate or sustain reprogramming to pluripotency and terminate in states with altered non-pluripotent identities.

## Stoichiometry and temporal dynamics of OSKM expression drive diverse cell fate trajectories

The absolute and relative stoichiometry of ectopic OSK at initiation and over the overexpression time course have profound effects on the progression and efficiency of reprogramming (Papapetrou *et al.*, 2009; Carey *et al.*, 2011; Yamaguchi *et al.*, 2011; Takahashi and Yamanaka, 2016; Buganim *et al.*, 2013). At the single-cell level, several factors including differential efficiency of Sendai virus delivery, differences in antiviral response and natural stochasticity, could result in differential combinatorial expression of mRNA and protein levels of OSKM. In our system, we reprogram cells using 3 vectors: *KLF4*, *MYC*, and a polycistronic KOS vector (Fig 1a). We would hence expect cells to express factors deriving from combinations of these vectors. The inherent stochasticity of transgene expression and its temporal dynamics sets up a natural experiment that allows us to dissect cell states and fates associated with different doses of the reprogramming TFs over the course of reprogramming.

We observed systematic differences in temporal dynamics of total scRNA-seq expression levels of OSKM as well as their deconvolved endogenous and exogenous levels across cell state transitions (Fig 2a,b, Fig S2a, Methods). The broad patterns of variation of total OSKM expression were also reflected in their ChromVAR deviation scores, a surrogate score for binding levels of each of the TFs based on the scATAC-seq accessibility at peaks containing their canonical motifs (Fig 2c, Fig S2b,c,d Methods) (Schep *et al.*, 2017). The expression and ChromVAR deviation scores across cells correlated strongly for OSK but not *MYC* (Fig S2c, Spearman correlation 0.70, 0.53, 0.51, −0.06 for *OSKM* respectively), which is likely due to *MYC*’s inability to directly initiate chromatin accessibility in contrast to the *OSK* pioneer TFs (Soufi *et al.*, 2012; Chronis *et al.*, 2017).

To quantitatively understand how the combinatorial expression of OSKM varied across the reprogramming time course, we clustered cells using *k*-means (*k*=8) based on single-cell expression levels of OSKM (Fig 2d, Fig S2a,b, Methods). While fibroblasts, as expected, expressed very low or no transcripts of OSKM, distinct patterns of OSKM expression in cells day 2 and beyond were associated with different outcomes. We first analyzed the off-target trajectories (T1, T2). Beyond day 0, the majority of fibroblast-like cells in clusters C2-C5 along trajectory T1 expressed either low levels of OSKM or elevated levels of *MYC* only. The population of keratinocyte-like cells (C6) along trajectory T2 expressed high levels of KM but not OS, reminiscent of a *KLF4* driven keratinocyte-like off-target cell state found in scRNA-seq analysis of mouse reprogramming (Guo *et al.*, 2019). *KLF4* has been implicated in epidermal development (Segre *et al.*, 1999), and expression of *KLF4* alone in mouse embryonic fibroblasts (MEFs) can induce a keratinocyte-like fate (Guo *et al.*, 2019).

We next analyzed cells on the reprogramming (T3) and partially reprogrammed (T4) trajectories. Cells that seemed to initiate reprogramming at day 2 express higher levels of all 4 factors, with *OCT4* expression levels exceeding those of the other factors (Fig S2a,b). Post OSKM induction, day 2 cells split into either hOSK (C7, median expression across cells for OSKM 1863, 583, 893, 1023 TPM respectively) or xOSK (C8, OSKM expression 7217, 2194, 2440, 1196 TPM respectively) states (Fig S2a). Median expression levels for day 2 cells in the xOSK state were 4-fold higher for *OCT4* and 8-fold higher for *SOX2* compared to iPSC levels (*OCT4*: 1740 TPM, *SOX2*: 271 TPM, negligible KM ~ 0 TPM) (Fig S2a).

While premature withdrawal of OSKM expression can stall the progress of cells in a partially reprogrammed state (Tanabe *et al.*, 2013; Polo *et al.*, 2012), eventual silencing of transgenes is required for maturation of Pre-iPSC cells (Golipour *et al.*, 2012). OSKM transgene expression levels gradually attenuated over the time course, owing to dilution of Sendai virus vectors (Fusaki *et al.*, 2009). Compared to day 2 cells in xOSK state, day 10 cells in the Intermediate state C12 along the reprogramming trajectory T3 were down nearly 10-fold for OSK (OSKM expression 833, 175, 176, 750 TPM respectively, Fig S2a) and the final population of iPSCs was transgene-free. Transgene expression levels were higher along states on the primary reprogramming trajectory (T3) compared to the partially reprogrammed trajectory (T4) (OSKM expression 305, 77, 64, 522 TPM respectively for day 10 cells in Intermediate state C9).

In summary, we observe that cells with reprogramming potential express supraphysiological levels of all 4 reprogramming factors, many folds higher than in iPSCs, followed by a gradual reduction across the time course. Further, sustained expression of reprogramming factors at or beyond their levels in iPSCs may be critical to avoid partial reprogramming (Ilia *et al.*, 2023). Insufficient expression of any one of the transgenes results in off-target cell fates or failure to initiate reprogramming. Together, these results support a strong association between dynamics of OSKM stoichiometry and reprogramming outcomes.

## Base-resolution deep learning models reveal cis-regulatory sequence codes of cell-state resolved chromatin accessibility landscapes

The stoichiometric overexpression of OSKM in fibroblasts triggers a cascade of changes in expression of other TFs. TFs combinatorially bind constellations of DNA sequence motifs encoded in regulatory elements to modulate their chromatin state and target gene expression (Sridharan *et al.*, 2009; Knaupp *et al.*, 2017; Chronis *et al.*, 2017; Soufi *et al.*, 2012, 2015). A quantitative understanding of how cis-regulatory sequence influences TF binding and chromatin accessibility dynamics across diverse reprogramming fates has been elusive due to lack of high-resolution data and the combinatorial complexity of the cis-regulatory code.

**Fig 3:**
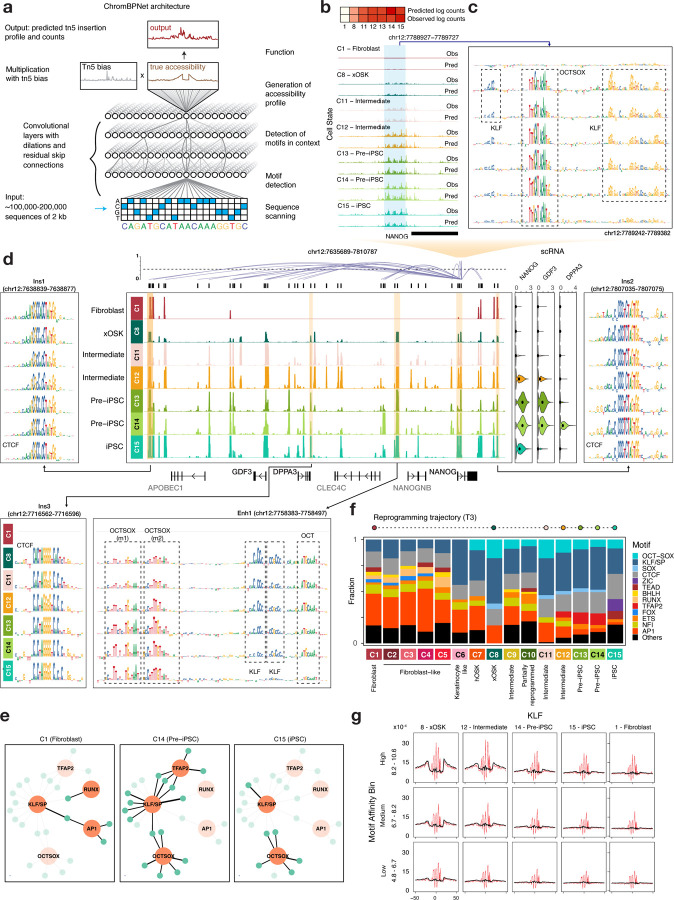
Base-resolution deep learning models to learn cell-state specific cis-regulatory sequence code **a)** ChromBPNet model architecture: A single ChromBPNet model predicts the base-resolution pseudo-bulk scATAC-seq signal for a given cell state for a 2kb input sequence, while correcting for Tn5 sequence bias **b)** Normalized counts prediction of ChromBPNet models for each of the cell states along the primary reprogramming trajectory (T3) at the *NANOG* promoter (chr12:7788327-7790327) (top) and per base predictions over the central 1kb (bottom) **c)** Model-derived cell state-specific contribution scores directly upstream of the *NANOG* promoter. **d)** Pseudo-bulk scATAC-seq read pileup in the 176kb domain containing *NANOG* (chr12:7635000-7811000), along with expression of *NANOG*, *GDF3* and *DPPA3*, and peak-gene links for *NANOG* (center). Contribution reads for selected peaks within the domain. Contribution scores are shown only for cell states for which the locus was called as a peak. **e)** TF-to-gene network of NANOG: motifs are represented by orange nodes and peaks by blue nodes. Motif nodes are on when the motif is learned by the model for that cell type, and peak nodes are active if the peak is accessible in the cell type. An edge is active if an instance of the motif is found in the peak. **f)** For each cell state, the relative proportion of predictive instances of prominent motifs out of all predictive instances. **g)** Virtual footprinting for KLF obtained by inserting instances of KLF stratified by log-odds scores into random background sequences and averaging predicted profile probability distributions with (red) and without (black) bias for each cell state. States are ordered by decreasing KLF4 concentration.

To decipher the cis-regulatory sequence code of accessible regulatory elements in the 15 landmark cell states across the reprogramming time course, we trained convolutional neural network models called ChromBPNet. ChromBPNet learns sequence pattern detectors akin to TF binding motifs and their higher-order organizational syntax from DNA sequence to predict base-resolution, pseudo-bulk scATAC-seq coverage profiles in 2kb windows around scATAC-seq peaks and in background regions from each cell state (Fig 3a, Methods) (Pampari *et al.*, in preparation). ChromBPNet jointly models the total Tn5 insertion counts and their distribution (shape of the profiles) across these windows at single base resolution, as a function of the underlying sequence, while regressing out biases due to Tn5’s intrinsic sequence preference. We used a 10-fold, chromosome hold-out cross-validation scheme to train, tune, and evaluate the predictive performance of the models. We obtained high and stable Pearson correlation between total predicted and observed Tn5 insertion coverage in test regions across all folds and cell states (Fig S3a,b). The observed and predicted base-resolution distributions of Tn5 insertions (shapes of the profiles) in test peak regions were also concordant (Fig S3c,d). For example, ChromBPNet models accurately recover the changes in magnitude and shape of accessibility profiles at the *NANOG* promoter (chr12:7788327-7790327) as it gains accessibility across the reprogramming time course (Fig 3b).

We used the DeepLIFT algorithm to derive the quantitative contribution of each base in any regulatory sequence of interest to its corresponding predicted accessibility profile from each cell-state-specific model (Fig 3c) (Lundberg and Lee, 2017; Shrikumar *et al.*, 2017). At the *NANOG* promoter sequence, DeepLIFT reveals bases overlapping an evolutionarily conserved, high-affinity OCT-SOX binding motif gaining predictive contribution in the earliest time point after induction (xOSK state) and retaining high contribution through the time course, including the iPSC state (Rodda *et al.*, 2005). In contrast, a constellation of adjacent KLF binding motifs first engaged in the xOSK state, show diminishing contributions through the time course with a complete loss in the iPSC state, which corresponds with the lack of *KLF4* expression in fibroblasts and iPSCs (Fig S2a,b). Hence, contribution scores derived from cell-state-specific ChromBPNet models can be used to infer putative bound TF motif instances that influence chromatin accessibility and also track putative TF binding occupancy dynamics across the time course in terms of motif contributions to chromatin accessibility.

We used our model-derived contribution scores to further explore the sequence basis of chromatin accessibility dynamics of all scATAC-seq peaks in the ~176kb domain containing the *NANOG* gene (Fig 3d, Fig S4a). This domain is largely inaccessible in fibroblasts and is flanked by two constitutively accessible peaks (Ins1 and Ins2) containing CTCF motifs with ubiquitously high contribution scores, which overlap loop anchors in micro-C contact maps in fibroblasts and ESCs (Fig S4b) (Krietenstein *et al.*, 2020). A putative enhancer element (Enh1) ~30 kb upstream of the *NANOG* TSS is one of the earliest to gain accessibility in the xOSK state as a likely early target of OSK over expression. The contribution scores support this hypothesis by highlighting an array of OCT-SOX, KLF and OCT motifs which are predicted to open chromatin post-induction (xOSK state) and maintain accessibility throughout the time course post-induction. The KLF motifs once again show diminishing contributions over the time course with no contributions in the iPSC state, which is consistent with decreasing *KLF4* expression across the time course and eventual loss in iPSCs (Fig S2a). Four days into the reprogramming time course (C11 - Intermediate state), an element (Ins3) downstream of the *DPPA3* gene gains accessibility via a CTCF motif with a high contribution score, remaining accessible up to and including the iPSC state. This element overlaps a loop anchor of a sub-domain that is observed in micro-C data only in H1-hESCs but not fibroblasts (Krietenstein *et al.*, 2020) (Fig S4b). Soon after the gain of this loop anchor and likely formation of the sub-domain, *NANOG* expression rapidly increases in concert with increasing accessibility of multiple putative enhancers linked to *NANOG* within the larger domain.

We constructed cell-state-specific TF-to-gene (TF2G) networks regulating *NANOG* by summarizing the TF binding sites with high contribution scores in all dynamic accessible peaks linked to *NANOG* across the reprogramming cell states (Fig 3e, S4c Methods). These dynamic TF2G networks implicate OSK and somatic TFs RUNX and AP-1 driving chromatin dynamics in the *NANOG* locus during early reprogramming cell states (C8, C11, C12). TFAP2 is predicted to transiently co-occupy accessible sites primarily with KLF in the Intermediate (C12) and Pre-iPSC states (C13, C14). *NANOG* expression is highest in the Pre-iPSC states (C13, C14) which correspond to extensive co-occupation of putative enhancers by OSK and TFAP2.

Analogous dissection of the cis-regulatory sequence code and TF2G networks of the locus around the fibroblast-specific gene *FN1* implicates somatic TFs AP-1, RUNX and TEAD as its primary regulators in fibroblasts. On induction of OSKM, the *FN1* locus rapidly loses accessibility at multiple enhancers and by Day 4 its expression is reduced more than 10 fold (Fig S5a,b). Inferred TF2G networks regulating *JUN*, a member of the AP-1 subunit, implicate motifs of TEAD and KLF/SP factors as well as autoregulatory feedback involving gradual loss of AP-1 dependent enhancers over the course of reprogramming (Fig S5c) (Angel *et al.*, 1988). Thus, ChromBPNet enables intricate, multi-scale dissection of the cell-type resolved, dynamic cis-regulatory sequence code at the resolution of individual base-pairs, elements and regulatory domains of genes.

We expanded our analysis genome-wide, by deriving base-resolution contribution scores for all cell-state resolved scATAC-seq peaks from corresponding ChromBPNet models. While high contribution sequence patterns in peaks often resembled known canonical motifs of TFs, we occasionally observed significant deviations. For example, a high contribution score motif instance (m2) in the earliest Nanog enhancer (Enh1) resembles a partial version of known OCT-SOX motifs and scores poorly as a sequence match to any known motif from multiple motif databases (Vierstra *et al.*, 2020) (Fig 3d). Hence, to enable *de novo* discovery of refined and novel motif patterns, we used the TF-MoDISco algorithm to cluster and consolidate subsequences with high contribution scores across all peaks from each cell state into non-redundant motif patterns (Shrikumar *et al.*, 2018). TF-MoDISco identified 30 non-redundant, *de novo* motifs across all 15 cell states (Fig S6a). We scanned the contribution weighted sequences of all peaks across all cell states with the TF-MoDISco motifs to obtain a comprehensive annotation of predictive motif instances across the genome and summarize variation in the number of predicted motif instances across the cell states (Fig 3f, Methods). In Fibroblasts (C1), AP-1 and CTCF are the most prominent predictive motifs within peaks, followed by NFI, BHLH, SP and FOX motifs. Post OSKM induction, the accessible chromatin landscape is dominated by OCT-SOX, SOX and KLF motifs in the xOSK (C8) state which account for >50% of all predictive motif instances recovered from peaks. In successive states, the fraction of OSK motifs reduces in tandem with a withdrawal of OSK expression levels. TFAP2 motifs transiently drive accessibility in Pre-iPSC cells (C13, C14). ZIC and TEAD motifs gain prominence in the iPSC state (C15), while CTCF motifs increase to account for nearly 24% of all motif instances. These results suggest that chromatin dynamics in reprogramming is encoded in a compact, combinatorial and dynamic TF motif lexicon.

## Transcription factor concentration and motif affinity influence scATAC-seq footprints across reprogramming

TF binding at cognate DNA motifs can render the bound nucleotides refractory to Tn5 transposition. Analyzing Tn5 transposition pile-ups at nucleotide resolution can reveal these TF “footprints”, thereby providing additional support for TF occupancy at motif instances (Sung *et al.*, 2016). However, TF footprinting with ATAC-seq is particularly challenging due to Tn5’s strong sequence preference which can significantly distort the observed base-resolution coverage profiles and obfuscate the underlying latent TF footprints (Li *et al.*, 2019; Bentsen *et al.*, 2020). For scATAC-seq, the problem is compounded by shallow read depth for individual cell states often composed of a sparse number of cells. ChromBPNet models learn the relationship between DNA sequence and observed base-resolution ATAC-seq profiles while accounting for Tn5 sequence preference bias. Hence, we can use the model to regress out Tn5 bias and impute bias-corrected, de-sparsified latent profiles at single base resolution for any genomic or synthetic sequence. For example, at the CTCF bound loop anchor (Ins2) in the *NANOG* locus, the uncorrected predicted scATAC-seq profiles closely match observed scATAC-seq profiles across all cell states and show a strong footprint over the CTCF motif (Fig S7a, Fig 3d). However, the bias-corrected predicted profiles differ substantially from the observed and uncorrected profiles, highlighting a deeper, narrower footprint with increased protection from Tn5 transposition right over the CTCF motif. The spurious read profiles that are eliminated in the bias corrected profile closely resemble the predicted Tn5 bias profile for this sequence (Fig S7a). The characteristic depth and shape of TF footprint profiles at canonical motif instances have been previously shown to be a function of residence time of the TF (Sung *et al.*, 2016). CTCF has a high residence time (on the order of minutes) which is consistent with its motif instance being highly protected from Tn5 transposition (Nakahashi *et al.*, 2013; Vierstra *et al.*, 2020).

We performed a complementary marginal footprinting analysis using synthetic sequences to corroborate our observation at this locus. We embedded sequences with the highest matching score (surrogate for affinity) to the learned CTCF motif in a library of inaccessible GC-matched background sequences randomly sampled from the genome (Fig S7b, top row). We then used the ChromBPNet models to predict uncorrected and bias-corrected scATAC-seq profiles for the library and averaged the profiles over the entire library to derive marginal uncorrected and bias-corrected CTCF footprint profiles. We observed highly consistent uncorrected and corrected marginal footprints over the highest affinity CTCF motif across all cell states despite being derived from independently trained ChromBPNet models, suggesting robust predictions and reproducible bias correction. The corrected footprints once again showed stronger protection at the CTCF motif by eliminating Tn5 bias. We then derived marginal footprints using synthetic sequence libraries containing single CTCF motif instances sampled over a wide range of affinities (Fig S7b, Methods). We observed substantial and consistent attenuation of footprint strength with decreasing motif affinity across all cell states.

Next, to systematically study the influence of motif affinity and TF concentration on scATAC-seq footprints, we predicted a comprehensive constellation of marginal bias-corrected footprints for all 30 TF-MoDISco motifs over a range of sampled motif affinities binned into 3 strata (high, medium and low affinity), using ChromBPNet models from the 15 cell states which exhibit dramatic changes in TF concentrations across the reprogramming time course (Fig X3). The marginal footprints across the 30 motifs exhibited diverse and distinct characteristics. Several motifs showed systematic trends in properties of marginal footprints as a function of TF concentration across cell states and motif affinity across affinity strata.

For KLF motifs, the predicted marginal profiles showed footprints with stronger protection to Tn5 tagmentation after bias correction. For each affinity stratum of embedded KLF motifs, the strength of marginal footprints tracks expression of *KLF4* across cell states (Fig 3g). Similarly, for each cell state, marginal KLF footprints get stronger with increasing motif affinity. The footprints are strongest in the xOSK cell state. Even the weakest affinity motif stratum shows detectable footprints. In contrast, the weakest affinity motifs do not generate footprints in Pre-iPSCs at intermediate levels (~80 TPM) of *KLF4* expression. iPSCs and fibroblasts, which have trace levels of *KLF4* expression, do not show footprints across the affinity spectrum including the strongest motif instances (Fig S2a).

Sox2 binds DNA with short residence times (~11 seconds) with a marginal increase (~15 seconds) in the presence of Oct4 (Chen *et al.*, 2014). Previous reports have attributed the lack of average DNase-I footprints at SOX2 motifs in ChIP-seq peaks to its short residence time (Sung *et al.*, 2014, 2016). However, ChromBPNet models predict shallow but detectable marginal footprints at SOX and OCT-SOX motifs exclusively in the hOSK and xOSK state, suggesting that TFs with short residence times may in fact present ATAC-seq footprints at very high concentrations. (Fig S7c,d).

The somatic factor AP-1 also shows a strong relationship between marginal footprint strength, expression and motif affinity. The footprints are the strongest in fibroblasts and weakest in iPSCs. Weak affinity sites exhibit detectable footprints in fibroblasts which are nearly abolished in the xOSK state after OSKM induction (Fig S7e). The in-silico marginalization experiments are consistent with aggregation of observed Tn5 insertions over actual motif instances (Fig S7f,g,h).

These results demonstrate that ChromBPNet models trained on scATAC-seq data are able to reveal the intricate relationship between TF residence times, motif affinity and concentration on chromatin accessibility landscapes within and across cell states at unprecedented resolution.

## Peak sets of coordinated chromatin dynamics are regulated by distinct dynamic regulatory syntax

**Fig 4:**
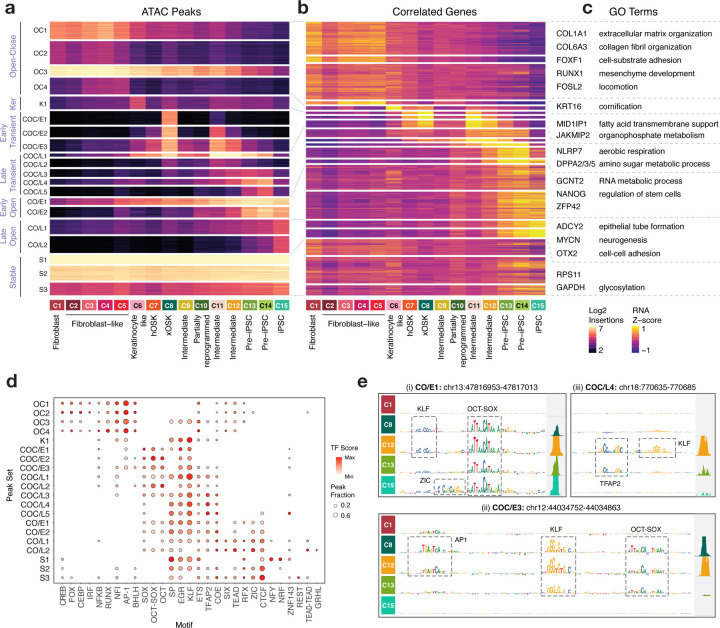
Monotonic and transient chromatin peak sets **a)** Normalized accessibility of peaks across cell states grouped into 20 peak sets, further grouped into 7 major archetypes **b)** Normalized gene expression of genes linked to each of the peaks in each peak set **c)** Representative genes and gene set enrichment terms summarized for each peak set archetype **d)** Motif activity scores and abundances for each peak set **e)** ChromBPNet contribution scores and observed accessibility signal at 3 representative loci

Overexpression of OSKM dramatically reconfigures the chromatin landscape of fibroblasts. For example, ~56% of the scATAC-seq peaks in the xOSK cells that first emerge 2 days post induction and anchor the primary reprogramming trajectory (T3) are not accessible in fibroblasts. An equivalent proportion of fibroblast peaks are closed in the xOSK state (Fig S8a). Previous studies have shown that OSKM orchestrate reprogramming by engaging promoters and distal cis-regulatory elements that initiate a cascade of chromatin remodeling events and corresponding transcriptomic changes (Deng *et al.*, 2021; Chronis *et al.*, 2017; Knaupp *et al.*, 2017; Li *et al.*, 2017). Hence, to identify modules of open chromatin regions with synchronized dynamics, we first identified 71 clusters of cells representing higher resolution subpopulations across the entire time course (Fig S8b, Methods). We then clustered the 525,835 scATAC-seq peaks from the 15 cell states based on their accessibility dynamics across the 71 clusters to obtain 20 peak sets with distinct patterns of accessibility, followed by summarization of their accessibility over the 15 cell states (Fig 4a). The 20 peak sets were further grouped into seven major archetypes of chromatin accessibility dynamics across the time course. To identify target genes potentially regulated by these dynamic peaks, we linked peaks to genes based on pairwise correlation of their scATAC-seq and scRNA-seq respectively across the time course (Fig 4b, Methods). We performed a gene set enrichment analysis on genes linked to each peak set to investigate their putative functional roles (Fig 4c, Table S2, Methods). To understand cis regulation of these dynamic peak sets, we quantified global TF motif activity scores for each peak set by consolidating predictive instances of all 30 TF-MoDISco motifs in their respective peaks across all cell states (Fig 4d, Methods).

We first considered peak sets with distinct monotonic accessibility patterns over the course of reprogramming. The Open-Close peak set (OC1-4), consists of 148,901 fibroblast-specific peaks that close over the reprogramming time course. 8386 genes linked to OC peak sets are enriched for fibroblast terms including collagen formation and extracellular matrix assembly. OC peaks are predicted to be regulated by AP-1, ATF, CEBP, RUNX, IRF1, NFI, and FOX motifs.

Close-Open peak sets consist of iPSC-specific peaks closed in fibroblasts that gained accessibility either early (CO/E1-2, 38875 peaks) or late (CO/L1-2, 70112 peaks) during reprogramming. 8118 genes linked to Close-Early Open (CO/E) peaks are involved in stem cell regulation and RNA metabolic process, while 3860 genes linked to Close-Late Open (CO/L) peaks are enriched for cell-cell adhesion and neurogenesis processes, consistent with previous analyses (Cacchiarelli *et al.*, 2015). CO/E and CO/L peaks are primarily regulated by OCT-SOX and KLF motifs as well as CTCF, TEAD, GRHL and ZIC motifs. However, different combinations of motifs are engaged at different time points across the time course via cooperative binding of OSK with other TFs that are expressed at specific time points. For example, at the CO/E1 peak chr13:47816815-47817315, the early gain of accessibility in the xOSK state (C8) is attributed to OCT-SOX and KLF motifs. Over the course of reprogramming, contribution of the KLF motif diminishes while a predictive ZIC motif instance emerges in the iPSC state, concomitant with late expression of multiple *ZIC* TFs (Fig 4e (i), Fig X2d), in line with the late stage ‘enhancer selection’ model proposed in Chronis *et al.* (Chronis *et al.*, 2017).

We next focused on transient peaks that are closed in both fibroblasts and iPSCs, but accessible in various intermediate cell states along the primary reprogramming trajectory. Early Transient peaks (COC/E1-3, 81255 peaks) are closed in fibroblasts but open at the first time point after OSKM induction in the xOSK (C8) state, before closing eventually in iPSCs. COC/E peaks are enriched primarily for OSK motifs, suggesting that they arise as an immediate consequence of OSKM overexpression. This reinforces the idea that OSK can act as pioneer factors and recognize their cognate motifs on nucleosomal DNA and open closed chromatin (Soufi *et al.*, 2012, 2015). In addition, predictive instances of AP-1 motifs also feature in Early Transient peaks, consistent with previously observed redistribution of somatic TFs to transient sites (Fig 4d, e(ii)) (Chronis *et al.*, 2017; Knaupp *et al.*, 2017; Deng *et al.*, 2021). Since *OCT4* and *SOX2* are constitutively expressed in all cell states from xOSK through iPSCs, and *KLF4* is also expressed till day 14 Pre-iPSC (C14) cells (Fig S2a), the loss of COC/E peaks a few days after their opening strongly hints at stoichiometry-dependent action. Pseudotime analysis of TF expression and motif activity as measured by ChromVAR along the reprogramming trajectory (T3) (Fig S8c,d, Methods) further suggests expression dependent binding activity of OSK and AP-1. Notably, 1913 genes linked with all Early Transient peak sets (COC/E) show a marked lack of gene-set enrichment relative to other peak sets, consistent with previous studies (Table S2, Fig S9a) (Cacchiarelli *et al.*, 2015). We explore these COC/E peaks in more detail in the next few sections.

Late Transient peaks (COC/L1-5, 77431 peaks) are also closed in fibroblasts, but attain maximum accessibility among reprogramming cell states between days 4–14, and then close in iPSCs. COC/L peaks likely arise due to secondary effects of OSKM overexpression. In contrast to Early Transient peaks that primarily show OSK and AP-1 binding, Late Transient (COC/L) peaks are relatively depleted for O binding but displayed high scores for TFAP2 motifs (Fig 4d, e (iii)). *TFAP2C*, which is a pioneer factor, is the only *TFAP2* family TF expressed during reprogramming (Fig X2d). COC/L peaks are linked to pre-implantation genes such as *DPPA2/3/5* and *DNMT3L* that are preferentially expressed in Pre-iPSC cells (Fig X2d). This is consistent with the observation that elevated levels of *KLF4* in Pre-iPSCs relative to iPSCs activate *TFAP2C* to drive cells to a transient naive, pre-implantation-like state (Liu *et al.*, 2020; Pastor *et al.*, 2018; Tan *et al.*, 2011; Cacchiarelli *et al.*, 2015; Gafni *et al.*, 2013; Takashima *et al.*, 2015). The chromatin accessibility landscape of Pre-iPSC cells resembles a hybrid naive-primed state (Fig S9b). The hybrid naive-primed state likely facilitates media-dependent exit into either naive or primed iPSC state.

Additionally, a set of keratinocyte-specific peaks (K1, 28388 peaks) is highly accessible in the keratinocyte-like population (C6), linked with 781 genes including keratinocyte markers such as *KRT16* and *KRT14*, and enriched for the GO term cornification. As expected, Keratinocyte-like specific peaks (K1) are enriched primarily for KLF motifs but not OCT-SOX. Finally, constitutively accessible peaks (Stable S1-3, 80873 peaks) are localized near both fibroblast and iPSC genes, as well as housekeeping genes such as *GAPDH* and *RPS11*.

Thus, overexpression of OSKM triggers a dramatic, multi-phasic remodeling of chromatin accessibility and gene expression involving 444,962 dynamic scATAC-seq peaks linked to 12,135 dynamic genes including 1074 TFs. Consistent with previous analyses, while some iPSC-specific peaks are gained early during reprogramming, the majority are acquired during the late stages of reprogramming (Knaupp *et al.*, 2017; Li *et al.*, 2017; Liu *et al.*, 2020). Peak-centric analyses of cis-regulatory sequence highlight stoichiometry-dependent interplay of OSKM with somatic factors like AP-1, CREB, and RUNX, as well as TF families such as CTCF, TEAD and ETS that trigger the expression of their downstream targets. These downstream targets include other reprogramming TFs such as *TFAP2* and *ZIC*, that at later stages of reprogramming cooperatively bind with OSK to reinforce the pluripotency circuit. Beyond peaks with monotonic accessibility patterns, nearly 40% of all dynamic peaks along the primary reprogramming trajectory (T3) are transient, and drive poorly characterized gene expression programs.

## Early Transient peaks are unique to reprogramming and not found in differentiated cell types and tissues

**Fig 5:**
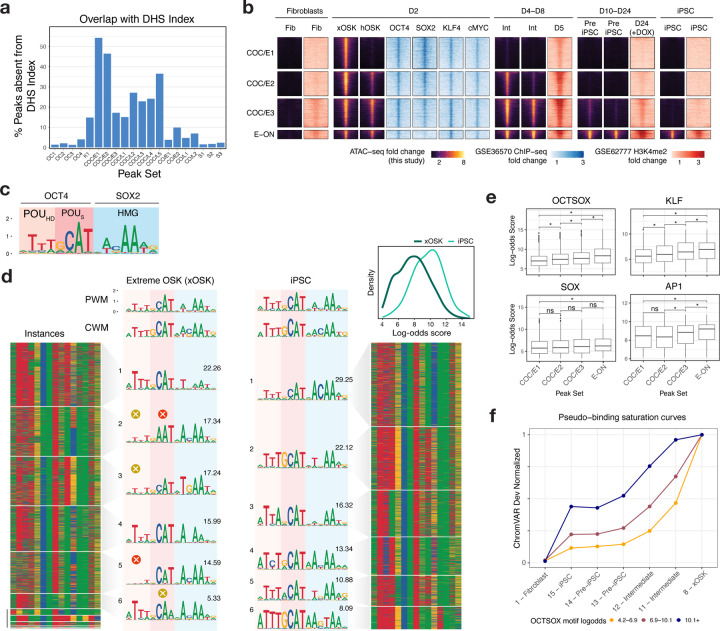
Characterization of Transient peaks sets **a)** Fraction of peaks in each peak set that are not present in a reference DHS Index of 438 biosamples **b)** Integration of scATAC-seq data with day 2 OSKM ChIP-seq data (GSE36570) and H3K4me2 ChIP-seq from fibroblasts, days 5, 24 and iPSC (GSE62777). 2kb regions are shown for scATAC-seq and OSKM ChIP-seq, and 4kb regions for H3K4me2 ChIP-seq **c)** Canonical OCT-SOX motif from (Avsec et al. 2021), labeled with its binding domains **d)** TF-MoDISco OCT-SOX motif recovered in the xOSK and iPSC cell states. Each row in the heatmap is one genomic instance of the OCT-SOX motif retrieved by TF-MoDISco. The instances are clustered into sub-motifs separately for each cell state by TF-MoDISco, with the relative fraction of each sub-motif indicated above its PWM. The inset shows the distribution of the log-odds scores over motif instances in the two cell types. **e)** Box-plot of the distribution of log-odds scores for motif instances of OCT-SOX, KLF, SOX and AP1 motifs within COC/E1-3 and E-ON peak sets, * *p*-value < 0.001 **f)** Pseudo-binding saturation curve for OCT-SOX. Each line is one strata of peaks split based on the affinity of the OCT-SOX motif/s present in them. States are ordered in roughly increasing order of combined OCT4 and SOX2 gene expression.

To decipher the role of the Early Transient peaks (COC/E), we first checked whether they overlapped accessible sites in a reference DNase I hypersensitive sites (DHS) index across 438 well characterized cell-lines, primary cells and tissues (Meuleman *et al.*, 2020) (Fig 5a, Methods). Nearly half of the peaks in Early Transient peak sets COC/E1 and COC/E2 are absent in all biosamples from the DHS index. In contrast, <5% of the peaks in Open-Close (OC1-3) and Stable peaks (S1-3) sets are absent in the DHS Index. The stark depletion of the early transient peaks in diverse cell types and tissues suggests these peaks may be a unique feature of our reprogramming time course.

To determine whether these early transient peaks are a general feature of reprogramming, we compared the Early Transient peak sets (COC/E1-3) and a contrastive set of peaks (E-ON) that opened early at Day 2 and remained accessible throughout our time course against OSKM ChIP-seq (Soufi *et al.*, 2012) and H3K4me2 histone ChIP-seq (Cacchiarelli *et al.*, 2015) (Fig 5b) data from two alternative reprogramming systems. Unlike our Sendai virus based reprogramming of dermal fibroblasts, Soufi et al. used doxycyline (dox)-inducible lentiviral transduction while Cacchiarelli et al. used secondary fibroblasts with constitutive human telomerase (hTERT) expression. All Early Transient and E-ON peak sets show strong enrichment of OSKM binding from ChIP-seq data at day 2 from (Soufi *et al.*, 2012). Moreover, H3K4me2 mirrors chromatin accessibility patterns of both peak sets across the time course (Cacchiarelli *et al.*, 2015). Hence, the early transient peaks are a reproducible signature of multiple OSKM induced fibroblast reprogramming systems.

## OCT/SOX target low-affinity non-canonical motifs at high initial concentrations to open chromatin in Early Transient peaks

Next, we investigated cis and trans regulatory factors that could potentially explain the gain and eventual loss of Early Transient peaks. Early Transient peaks primarily gain accessibility in the xOSK state (C8). OS expression is sustained over the course of reprogramming through iPSCs, and switches from exogenous to endogenous over the time course. However, the mRNA levels of *OCT4* and *SOX2* are 4-fold and 8-fold higher in xOSK compared to iPSCs (C15). Further, OSK motifs disproportionately dominate the chromatin landscape in the xOSK state (Fig 3f). These observations suggest that OSK are key regulators of chromatin accessibility dynamics of early transient peaks.

We suspected that supraphysiological concentration and stoichiometry of OS in the xOSK states could enable these pioneer factors to occupy low affinity, non-canonical binding sites resulting in esoteric landscapes of chromatin accessibility. Based on this hypothesis, we expected early transient peaks in the xOSK state to be enriched for non-canonical OCT-SOX binding sites.

To test this hypothesis, we first compared the *de novo* OCT-SOX motifs inferred by TF-MoDISco from ChromBPNet models of xOSK and iPSC states to a canonical OCT-SOX motif (Avsec, Weilert, *et al.*, 2021). The TF-MoDISco OCT-SOX motifs from xOSK and iPSC states were similar to the canonical OCT-SOX heterodimer motif which consists of three parts - a consensus subsequence ATTT followed by GCAT which are recognized by OCT4’s POU-homeodomain (POU_HD_) and POU-specific (POU_S_) domains respectively, and a trailing ACAA subsequence that binds to the SOX2 HMG domain (Fig 5c,d) (Reményi *et al.*, 2003). However, the xOSK-derived OCT-SOX motif showed subtle differences from the canonical motif in the two POU recognition subsequences. To obtain a deeper understanding of these differences, we identified all predictive motif instances of the xOSK and iPSC-derived TF-MoDISco OCT-SOX motifs from the xOSK and iPSC peaks respectively. We then computed the log-odds scores of the motif instances from xOSK and iPSCs against the canonical OCT-SOX motif as a surrogate measure of affinity. OCT-SOX motif instances from xOSK cells showed significantly lower log-odds scores compared to instances from iPSCs (Fig 5d, inset, Mann-Whitney *p*-value <2.2×10^−16^) (Methods), suggesting that the early transient peaks likely harbor low affinity OCT-SOX binding sites.

To reveal fine-resolution heterogeneity of OCT-SOX motifs in xOSK and iPSC peaks, we re-clustered the motif instances of the TF-MoDISco OCT-SOX motifs from each state into more homogeneous sub-motifs (Fig 5d). All OCT-SOX sub-motifs from iPSCs are similar to the canonical OCT-SOX motif in terms of engaging both POU domains and SOX-HMG. In stark contrast, several OCT-SOX sub-motifs from xOSK differed substantially from the canonical OCT-SOX motif. xOSK sub-motifs 2 and 6 show distinct suboptimal matches to the canonical recognition sequence (GCAT) of the POU_S_ domain. Further, in OCT-SOX sub-motifs 2,3 and 5, the leading ATTT subsequence that binds the OCT4 POU_HD_ domain is greatly attenuated, suggesting that a partial POU_S_-HMG heterodimer motif in transient peaks may be sufficient to recruit the OCT4-SOX2 heterodimer at high concentrations in the xOSK state. A recent study supports our hypothesis by showing that OCT4-SOX2 heterodimers can in fact bind nucleosome-embedded instances of this exact partial motif and drive chromatin accessibility (Michael *et al.*, 2020). Our results also complement ChIP-seq experiments that show that OSK can individually target their partial monomeric motifs at nucleosome-embedded sites early during reprogramming (Soufi *et al.*, 2015). Thus, our models suggest that the early transient peaks in OSK-induced early reprogramming are a result of unique occupancy patterns of OCT4 and SOX2 pioneer factors at supraphysiological concentrations at non-canonical low-affinity heterodimeric binding sites.

## Duration of transience correlates with motif affinity

The gradual loss of chromatin accessibility of early transient peaks from the xOSK state to iPSCs is associated with a smooth decrease in the concentration of the OSK factors. However, subsets of early transient peaks exhibit subtle differences in their decay rates across the time course. The COC/E1 subset of peaks lose accessibility immediately after the xOSK state at day 2. COC/E2 peaks remain accessible longer until intermediate cell state 11 (day 8). COC/E3 peaks exhibit the slowest decay and are accessible through intermediate cell state 12 (day 8) until the Pre-iPSC state (day 10). The variable decay rates across these peak subsets could be due to subtle differences in the affinities of non-canonical OSK motif instances in each of these subsets. To test this hypothesis, we compared log-odds scores of instances of the OCT-SOX, KLF and SOX motifs across the three subsets of early transient peaks. We further contrasted these early transient peak subsets against a control set of peaks (E-ON) that also gain accessibility at day 2 but remain accessible throughout the trajectory and in iPSCs (Fig 5e, Methods). For all three motifs, we observed significant differences in log-odds distributions between the four peak sets, such that peak sets with faster decay rates contained motifs with lower surrogate motif affinity.

Next, we stratified peaks from all four peak sets into three levels of OCT-SOX motif affinity based on the highest scoring OCT-SOX motif instances in each peak. For peak sets corresponding to each affinity stratum, we compared their ChromVAR deviation scores to *OCT4*/*SOX2* expression levels across all the cell states in the primary reprogramming trajectory. These surrogate binding saturation curves for OCT-SOX not only show that accessibility decreases with TF concentration but also that the decay rates are faster for peaks with lower affinity OCT-SOX motifs (Fig 5f, Methods). Surrogate binding saturation curves for *KLF4* showed similar trends (Fig S9c). Overall, our results suggest that early transient peaks gain accessibility in the xOSK state due to binding of OSK at supraphysiological concentrations to their motif instances across a wide range of binding affinity. The dynamic and variable rates of decay of accessibility across the time course can be attributed to progressive release of weaker affinity sites as TF concentrations gradually decline.

## Sequestration of AP-1 to transient sites is associated with somatic silencing at single-cell level

**Fig 6:**
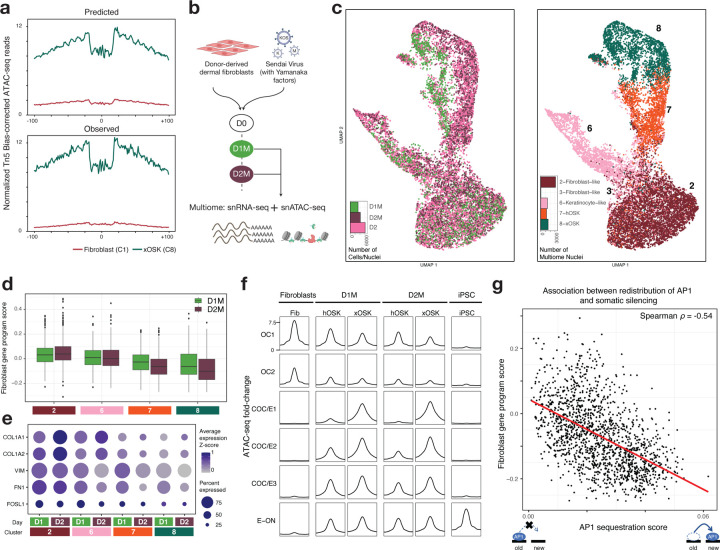
Single-nucleus multiome profiling highlights the role of Early Transient peaks in somatic repression **a)** Predicted (top) and observed (bottom) Tn5 bias-corrected footprints over AP1 motifs within Early Transient peaks in the xOSK state **b)** Schematic of experimental design **c)** UMAP of multiome cells integrated with D2 scATAC-seq cells labeled by sample (left) and cell state (right) **d)** Box-plot of single-nucleus expression of fibroblast-specific genes stratified by day and cell state **e)** Single-nucleus expression of representative fibroblast-specific genes stratified by day and cell state **f)** scATAC-seq and snATAC-seq normalized read pileup at OC, COC/E and E-ON peak sets **g)** Scatter-plot of AP1 sequestration score versus snRNA-seq expression of fibroblast-specific genes. Each point is a single nucleus from Day 2 multiome data from hOSK and xOSK states.

The role of early transient peaks in cellular reprogramming is not well understood. Previous studies have suggested that transient peaks are also bound by somatic TFs such as AP-1 and may play a role in redistributing somatic TFs away from fibroblast-specific peaks, thereby destabilizing the fibroblast chromatin landscape and promoting reprogramming (Chronis *et al.*, 2017; Deng *et al.*, 2021). Indeed, we find that the early transient peak sets are enriched for multiple predictive instances of somatic TF motifs, especially AP-1, alongside motifs of the OSK reprogramming factors (Fig 4d,e (ii)). Specifically, ~10,500 predictive AP-1 motifs supported by discernable ATAC-seq footprints are identified in peaks that first gain accessibility in the xOSK state (Fig 6a), a majority (~70%, i.e. ~7,500) of which are found in the early transient peak sets. Early transient peak sets with faster decay rates contain AP-1 motifs with lower surrogate affinity scores (Fig 5e). In contrast, ~35,000 (51%) of the ~69,000 predictive AP-1 motif instances found in fibroblast peaks are lost in the xOSK state.

We wondered whether there was an OSK dosage-dependent relationship between the displacement of somatic factors and repression of fibroblast gene sets. To study this phenomenon, we jointly profiled single-nucleus RNA-seq and ATAC-seq (sn-Multiome) at days 1 and 2 during the early stages of reprogramming (Fig 6b, Methods). We obtained 3217 nuclei from day 1 and 4161 nuclei from day 2 that passed quality control metrics (Fig X4, Methods). We annotated nuclei from the sn-Multiome data using label transfer from our previously annotated reprogramming map after harmonization and integration using the ATAC-seq modality (Fig 6c, S10a). All early cell states (Fibroblast-like (C2, 3), Keratinocyte-like (C6), hOSK (C7), and xOSK (C8)) from the original time course were represented in the multiome data.

We first analyzed snRNA-seq expression of fibroblast-specific genes in each of the cell states in the multiome data to gauge the extent of somatic silencing in the first two days of reprogramming. We estimated a “fibroblast gene program score” for each nucleus as the average expression z-score of fibroblast-specific genes (Fig 6d, Methods). Compared to fibroblast-like cells (C2), all other cell states showed reduced expression of fibroblast-specific genes within the first two days, consistent with transcriptional silencing of somatic targets during early reprogramming (Koche *et al.*, 2011). The xOSK state exhibited the lowest fibroblast gene program score followed by hOSK, both of which exhibited reduced expression of fibroblast-specific genes at day 2 relative to day 1. Key fibroblast marker genes including *COL1A1* and *FN1* showed reduced expression as early as day 1 (Fig 6e).

Analogous analysis of the snATAC-seq data showed that chromatin accessibility at fibroblast-specific peaks in the OC1 and OC2 peak sets decreased rapidly after OSKM induction. Compared to fibroblast cells at day 0, the xOSK state exhibited the lowest accessibility in OC1 and OC2 peaks followed by hOSK, with lower accessibility at day 2 than day 1 in both states (Fig 6f). Simultaneously, early transient peaks in the COC/E1-3 peak sets gained accessibility, with higher accessibility in xOSK state mirroring our previous analysis of scATAC-seq data (Fig 5b). The rapid loss and gain of accessibility at distinct sets of regulatory elements agrees with previously observed enhancer dynamics as early as before a single cell division upon overexpression of Yamanaka factors in MEFs (Koche *et al.*, 2011).

We then used the multiome data to test for quantitative association between the degree of sequestration of AP-1 to early transient peaks and repression of fibroblast-specific genes. For each nucleus, we estimated an “AP-1 sequestration score” as the proportion of ATAC-seq reads falling in Early Transient peaks containing predictive AP-1 motif instances relative to total reads in all peaks (Methods). Focusing on nuclei from hOSK and xOSK cell states at day 2, we observed a striking negative correlation (Spearman ⍴= −0.54, *p*-value < 2.2×10^−16^) between the fibroblast gene program score and the AP-1 sequestration score over early transient peaks (Fig 6g). This negative association holds after including fibroblast-like nuclei and also for nuclei from day 1 (Fig S10b,c). In contrast, an analogously computed “AP-1 retention score” aggregated across fibroblast-specific peaks containing predictive AP-1 motifs was positively correlated with fibroblast gene program scores across nuclei from hOSK and xOSK cell states at day 2 (Fig S10d, Spearman ⍴=0.59, *p*-value < 2.2×10^−16^). Together, these results suggest that overexpression of OSKM has a quantitative effect on somatic silencing in the early stages of reprogramming, mediated by redistribution of somatic TFs to newly opened regulatory elements.

## Discussion

Our study provides a comprehensive understanding of somatic cell reprogramming by combining single-cell data with an analysis of the interplay between temporal TF stoichiometry and motif syntax. Our single-cell RNA-seq and ATAC-seq atlas is a high-resolution and temporally dense profiling of reprogramming intermediates, in contrast to past efforts that sorted reprogramming intermediates based on surface marker expression (Polo *et al.*, 2012; Li *et al.*, 2017; Chronis *et al.*, 2017; Knaupp *et al.*, 2017; Cacchiarelli *et al.*, 2015; Xing *et al.*, 2020). The single-nucleus multiome profiling enriches the atlas with an unprecedented view into the earliest stages of fibroblast reprogramming. Using deep learning models, we generated a multi-resolution cell-state-specific map of regulatory elements. Analysis of this map yielded novel insights into the accessibility dynamics and sequence determinants of transient regulatory elements and their role in somatic cell gene silencing.

Our work showcases the potential of using bias-corrected, base-pair resolution neural networks to unravel stoichiometry-dependent low-affinity TF binding. In contrast to classical motif analysis methods such as PWM scanning, which suffer from low specificity when detecting low affinity TF binding sites, ChromBPNet learns *de novo* representations that highlight bases that quantitatively contribute to accessibility profiles without the need to specify arbitrary affinity thresholds. For a given TF, its relative contribution to accessibility drops with decreasing concentration, which is reflected both in the contribution scores and bias-corrected footprint shapes (Fig 3g). This enables a precise understanding of the dynamic occupancy of specific motif instances across the affinity spectrum and their impact on quantitative changes in chromatin accessibility within individual enhancers.

**Fig 7:**
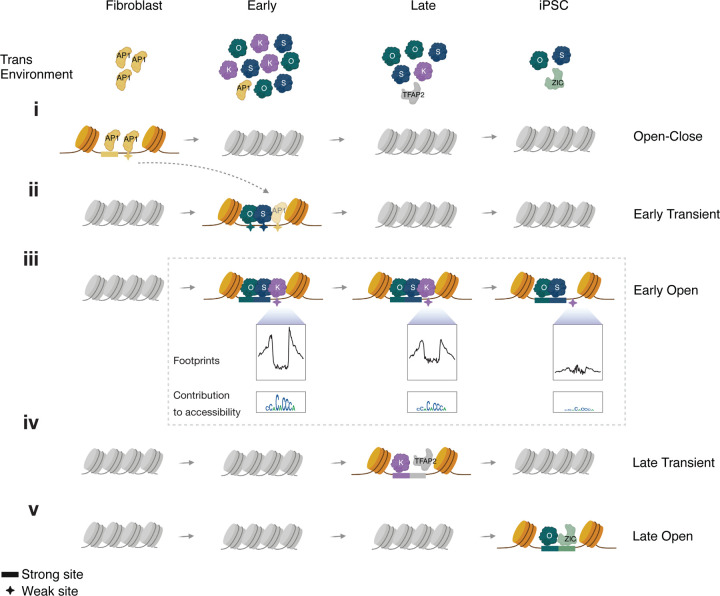
A refined model of reprogramming adapted from (Chronis et al. 2017)

OSKM combinatorially and quantitatively reconfigure the somatic cell state. Upon induction, stochastic variation in exogenous OSKM stoichiometry results in initial diversification into a putative reprogramming trajectory, and off-target keratinocyte-like and fibroblast-like fates. In cells with supraphysiological overexpression of all factors, OSK rapidly dominate the chromatin landscape by extensively binding to and opening closed chromatin, while fibroblast-specific peaks close early (Fig 7i–iii). Newly accessible sites primarily contain OSK motifs, which unambiguously supports the view that OSK act as pioneer factors upon overexpression (Knaupp *et al.*, 2017; Michael *et al.*, 2020; Li *et al.*, 2017). Intriguingly, a majority of sites that open early are lost over the course of reprogramming, concomitant with a reduction in the expression levels of OSKM. We show that Early Transient peaks are a reproducible signature of OSKM reprogramming and most of these peaks are exclusive to fibroblast reprogramming.. The gene expression program driven by early transient peaks is poorly characterized in terms of known and established gene sets. These findings suggest that extreme OSKM expression results in novel intermediate cell states distinct from well-characterized physiological states.

Within Early Transient regulatory elements, we find pervasive evidence of low-affinity binding sites of OSK, including an excess of partial POU_S_-HMG binding motifs. These observations suggest that the initiation of reprogramming is not tightly coupled to OSK binding specificity. This is supported by reports which show that substituting *OCT4* with mutant *OCT4* or orthologous *OCT6* in a reprogramming cocktail is able to initiate reprogramming in a manner similar to *OCT4* (Malik *et al.*, 2019; Roberts *et al.*, 2021). Further, we show that the accessibility at transient elements decays in an affinity-dependent manner such that the sites with weaker motifs close earlier (Fig 7ii), suggesting that reduced expression at least in part explains the silencing of transient elements. Our motif analyses also suggest that stage-specific expression of additional TFs determines subsequent chromatin dynamics. TFAP2C is primarily enriched in Late Transient peaks, which corresponds with a hybrid naive/primed Pre-iPSC state (Fig 7iv). Late Close-Open peaks, on the other hand, are enriched for motifs of ZIC and GRHL, suggesting that accessibility at these sites requires cooperativity of OS with iPSC-specific TFs (Fig 7v). Such intricate and granular analyses of stage-specific sequence drivers of chromatin accessibility and the footprints they generate underscores the importance of base-resolution deep learning models in the study of cis-regulation.

Our study provides new insights into the role of Early Transient peaks in somatic silencing. The ChromBPNet models revealed that Early Transient peaks harbor thousands of instances of AP-1 motifs, which was supported by the presence of discernable footprints (Fig 6a, 7ii). These findings are consistent with previous studies that have proposed a mechanism of “repression-by-theft”, wherein transient elements mediate the redistribution of somatic TFs away from their somatic targets (Chronis *et al.*, 2017; Knaupp *et al.*, 2017). This mechanism is analogous to the silencing of Runx1 targets caused by redistribution of Runx1 upon expression of the pioneer factor *SPI1* in T-cell development (Hosokawa *et al.*, 2018; Deng *et al.*, 2021), and the loss of pluripotency observed when introducing exogenous OCT4 binding arrays in ESCs (O’Hara and Banaszynski, 2022). The day 1 and 2 multiome analysis revealed a quantitative association between the sequestration of somatic TFs to newly opened sites and the repression of somatic gene expression. Our analyses suggest that this association is likely mediated by OSK stoichiometry, and may explain why sustained supraphysiological expression of the reprogramming factors is crucial for productive reprogramming. These findings not only enhance our understanding of reprogramming, but also have the potential to inform research on partial reprogramming, which involves the overexpression of reprogramming factors for a short interval of time (Puri and Wagner, 2023).

Together, this study provides a template for studying the effects of heterogeneous stoichiometry on cis-regulation. We exploit the stochasticity of Sendai virus induction to study the combinatorial state space of TF overexpression in reprogramming, and leverage powerful neural network models that learn cell state, TF stoichiometry-aware mappings from DNA sequence to chromatin accessibility. Our approach outlines a powerful paradigm for interrogating the stoichiometric and cis-regulatory sequence determinants of factor induced de-differentiation and trans-differentiation trajectories.

Our analysis is subject to certain limitations. First, the cell state transition trajectories identified in our study represent our best guess estimate by combining multiple lines of evidence including pseudotime inference, Sendai virus dilution, and findings from previous studies. Validating these trajectories would necessitate the use of lineage tracing methods. Second, the peak-gene linking is approximate, in part due to computational linking of scATAC-seq and scRNA-seq cells. This can impact the accuracy of the T2G networks. Third, ChromBPNet models explain chromatin dynamics that are predicted by local 2kb sequence windows. The models may be inaccurate when chromatin dynamics are determined by long-range interactions, and changes to methylation and histone modifications. For example, dynamic CTCF peaks that are closed in fibroblasts and open over the course of reprogramming are incorrectly predicted to be constitutively accessible by the models. To partially overcome this limitation, we restrict our analysis to peaks that are accessible in the respective cell types. Improvements in experimental, computational and modeling approaches will yield increasingly accurate and precise insights into the molecular mechanisms underlying cellular reprogramming. That said, our analytic approach should prove of broad utility and pave the way for new analyses and insights into nuclear reprogramming and TF behavior that may have previously been missed.

## Supplementary Material

Supplement 1

Supplement 2

Supplement 3

Supplement 4

Supplement 5

## Software and resources used

- ENCODE ATAC-seq and Chip-seq pipelines (Hitz *et al.*, 2023)
- ArchR (Granja *et al.*, 2021)
- 10x Genomics Cell Ranger 6.0.2 (Zheng *et al.*, 2017)
- GViz (Mathé and Davis, 2016)
- BentoBox (now PlotGardener) (Kramer *et al.*, 2022)
- SnapATAC (Fang *et al.*, 2021)
- Chromap (Zhang *et al.*, 2021)
- ggseqlogo (Wagih, 2017)
- logomaker (Tareen and Kinney, 2019)
- gProfiler2 (Raudvere *et al.*, 2019)
- cistromeDB (Zheng *et al.*, 2019)
- Seurat (Stuart *et al.*, 2019)
- DoubletFinder (McGinnis *et al.*, 2019)
- PAGA (Wolf *et al.*, 2019)
- Scanpy (Wolf *et al.*, 2018)
- Harmony (Korsunsky *et al.*, 2019)
- AMULET (Thibodeau *et al.*, 2021)
- ChromVAR (Schep *et al.*, 2017)
- ClusterR (Mouselimis *et al.*)
- DESeq2 (Love *et al.*, 2014)
- GimmeMotifs (Bruse and van Heeringen, 2018)
- Tomtom (Gupta *et al.*, 2007)
- HOMER (Heinz *et al.*, 2010)
